# Experimental Research on the Mechanical Properties of Tailing Microcrystalline Foam Glass

**DOI:** 10.3390/ma11102048

**Published:** 2018-10-20

**Authors:** Jinliang Bian, Wanlin Cao, Lin Yang, Cunqiang Xiong

**Affiliations:** 1 The College of Architecture and Civil Engineering, Beijing University of Technology, Beijing 100124, China; bjl211@163.com (J.B.); 18331083319@163.com (L.Y.); 2 Beijing North Glass Sinest Technology Co., LTD, Beijing 100125, China; 13901005122@139.com

**Keywords:** tailing microcrystalline foam glass, mechanical properties, constitutive model, experimental research

## Abstract

Tailing microcrystalline foam glass (TMFG) is a building material that not only has the characteristics of light weight, fire resistance, and thermal insulation, but also has decorative applications. TMFG has a broad application prospect, but there has been little research on the macroscale mechanical properties of this material. In order to analyze TMFG basic mechanical properties, a series of experimental studies were carried out by performing the four-point flexural, shear, uniaxial compression, and splitting tensile strength tests. The research showed that the foaming agent (SiC) had a great influence on the mechanical properties of the material. With the reduction of the amount of SiC, the strength of the material and brittle failure increased. The microcrystalline decoration surface improved the flexural strength and compression strength of the tailing microcrystalline foam glass. The modulus of elasticity and the Poisson’s ratio are discussed, and a formula for the modulus of elasticity is proposed. Based on the analysis of the stress and strain curves, a constitutive model is proposed for the application of tailing microcrystalline foam glass and future research on this material.

## 1. Introduction

Foam glass (FG) is a porous glass material composed of waste glass and various mineral waste materials. Generally, the production process of foam glass is as follows: Firstly, a uniform mixture is formed by adding appropriate amounts of the foaming agent, cosolvent, and various modified additives, and, then, the uniform mixture is put into a specific mold and subjected to preheating, melting, foaming, cooling, and other processes [1,2,3]. In 1935, the French company St. Gubain successfully developed foam glass using glass powder as the raw material and CaCO_3_ as the foaming agent; this mixture was heated in a refractory mold, to obtain a light material. St. Gubain developed a variety of silicate foam glass systems. Subsequently, the United States, Germany, Japan, the United Kingdom, and other countries also began the study of foam glass and issued a number of related patents and research reports. Foam glass has the advantages of low density, low thermal expansion coefficient, low thermal conductivity, and corrosion resistance. It enables heat insulation and sound absorption and is a lightweight, moisture-proof, and fire-proof building heat insulation material and decorative material. The development and application of foam glass will play a positive role in the recovery and utilization of glass wastes and in environmental protection [4,5,6,7].

**Figure 1:**
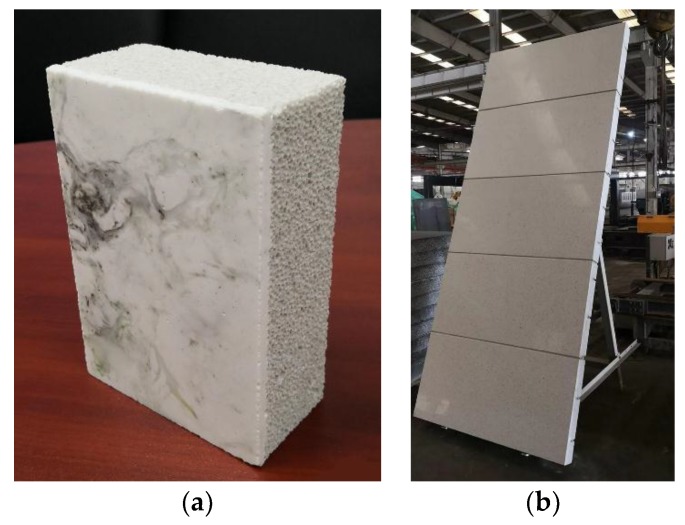
Tailing microcrystalline foam glass (TMFG). (**a**) TMFG with a microcrystalline decoration surface; (**b**) TMFG slab.

However, foam glass has a low mechanical strength, so it is rarely used as a wall material. Microcrystalline foam glass (MFG) produced by the micro-crystallization of foam glass is a lightweight, high-strength material. A large number of small bubbles and needle-like crystals are evenly distributed in the main phase of the glass. MFG has several advantages, such as being a fire-proof, non-toxic, non-radioactive, corrosion-resistant, and machinable material. Using MFG as a wall material can greatly reduce the weight of a building. Nowadays, tailing can produce not only MFG but also an MFG with a microcrystalline decorative surface by specific process, as shown in Figure 1. The material properties of foam glass, microcrystalline foam glass, and tailing microcrystalline foam glass (TMFG) are compared in Table 1 [8,9]. The compression strength of TMFG can reach 12.5 MPa, and its lowest thermal conductivity can reach 0.075 W/(m·K). The decorative surface is dense glass–ceramic, that can not only play a decorative role, but also greatly improve the mechanical strength of the material. TMFG can be used not only as a thermal insulation material but also to construct multi-functional exterior walls with integrated environmental protection, thermal insulation, fire protection, and decoration properties. Therefore, TMFG has a broad application prospect [10,11,12,13,14,15].

Fernandes H.R. [15] used recycled raw material as the main components to produce MFG, such as sheet glass cullet and fly ashes. He used SiC as the foaming agent and found that SiC led to apparent density and compressive strength values of about 0.18–0.35 g·cm^−3^ and 0.9–1.8 MPa, respectively. This research found that the compressive strength of MFG is affected not only by apparent density values, but also by the internal porosity and thickness of the struts and the crystalline phase composition. Chen B. [16] used a high content (50–70%) of fly ash to produce foam glass at a low temperature (800 °C). The foam glass exhibited great properties, such as low density and high porosity and mechanical strength (3.95 MPa). In past studies on FG, MFG, and TMFG, researchers focused on the raw materials and the preparation conditions [12,13,14,15,16,17,18,19,20,21], but there has been little research on the macroscale mechanical properties of TMFG. Since the macroscale mechanical properties are very important for the application of TMFG, a series of basic mechanical tests on the TMFG were carried out in this study: four-point flexural, shear, uniaxial compression, and splitting tensile strength tests. The basic mechanical properties and related constitutive models of TMFG were thus determined.

## 2. Materials

### 2.1. Batch Composition

The tailing microcrystalline foam glass (TMFG) used in the following tests was composed of quartz sand, waste stone residue, and glass. The batch composition was measured in terms of the weight percentage. The foaming rate was controlled by adding a foaming agent to adjust the strength and density of the TMFG. The main raw materials for all specimens were the same and were divided into three batch compositions. The specimens with microcrystalline decorative surfaces were based on the main raw materials with the addition of natural gravel. The specific batch compositions are listed in Table 2. 

### 2.2. Production Process

**Figure 2:**
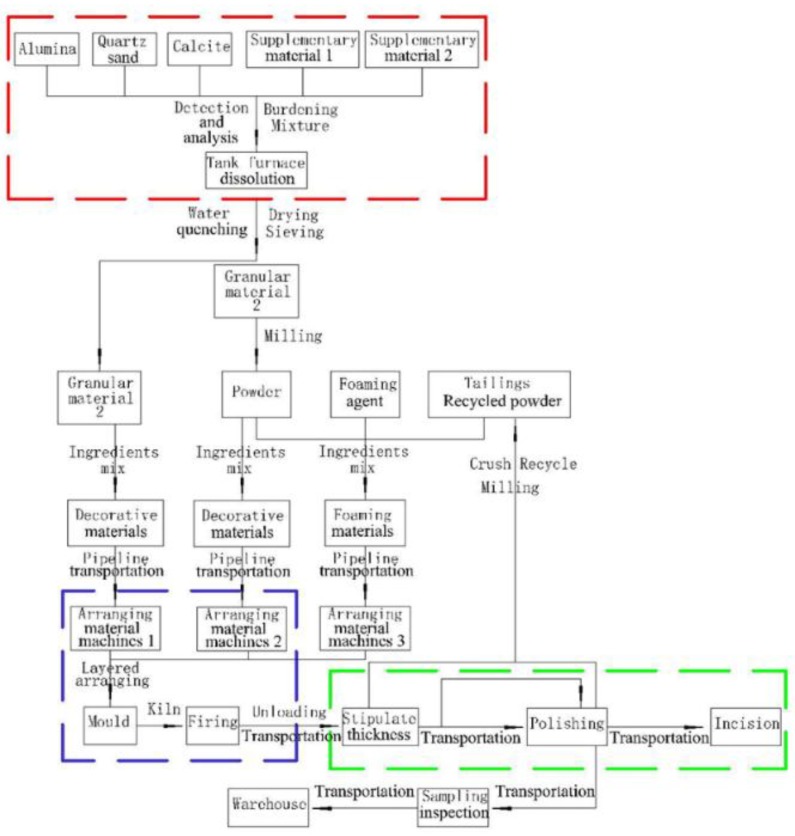
Production process of TMFG.

The entire production process of TMFG is shown in Figure 2. Firstly, the batch material was melted, and water was quenched using a tank furnace to obtain a basic glass raw material conforming to the requirements of crystallization. Then, the prepared material was placed into a mold and fired through a kiln. Finally, the product formed through polishing and cutting.

In Figure 2, the red, purple, and green dotted frames indicate the glass melting, sintering, and product processing processes, respectively. Thus, TMFG formed through three kinds of production processes.

### 2.3. Physical Properties of the Specimens

The bulk density, porosity, and water absorption of Group A specimens were tested. The test methods were adopted in GB/T 3810.3-2016 standard [22]. The size of the specimens was 100 mm × 100 mm × 100 mm (width × height × length). In each of the different tests, ten specimens per group were tested, and the results were averaged.

The specimens were placed in a drying box. The temperature was slowly raised to 110 ± 5 °C to dry the specimens to a constant mass, and then the specimens were moved to the dryer and cooled to room temperature. According to the criterion of constant mass, the quality of the specimen is weighed two times in 24 h at constant temperature, and the mass change rate is less than 0.1%. The dry weight (*m*_1_) of the specimens was determined.

A water tank made of inert material and a wooden grille with a section of about 50 mm × 50 mm were made. The size of the water tank should allow to immerse ten specimens. The specimens were placed on the wooden grille at the bottom of the water tank so that they were not in contact. Water was added to the water tank, and a 50 mm-thick layer of water was maintained above and below the specimens. The water was heated to boiling in 2 h, then the specimens were cooled in water to room temperature. Room temperature was maintained for 4 h. The treated specimens were placed gently in the hanging basket of an electronic balance and immersed in water to weigh their buoyant weight (*m*_2_).

The specimens were removed from the water, and the excess moisture was wiped off with a wet towel. The liquid in the air hole of the specimens was not sucked out. The saturated weight (*m*_3_) was immediately determined in air.

The bulk density, porosity, and water absorption of the specimens was calculated as follows:

(1) $$d=\frac{m_{1}}{m_{3}-m_{2}}$$

(2) $$P=\frac{m_{3}-m_{1}}{m_{3}-m_{2}}\times 100\%$$

(3) $$A=\frac{m_{3}-m_{1}}{m_{1}}\times 100\%$$

where d denotes the bulk density (kg/m^3^), P denotes the porosity (%), A denotes the water absorption (%), $m_{1}$ denotes the dry weight (kg), $m_{2}$ denotes the buoyant weight (kg), and $m_{3}$ denotes the saturated weight (kg).

The bulk density, porosity, and water absorption of groups A1, A2, and A3 are shown in Table 3. As can be seen from Table 3, the porosity and water absorption decreased with the increase of the bulk density.

## 3. Test Setup and Instruments

**Figure 3:**
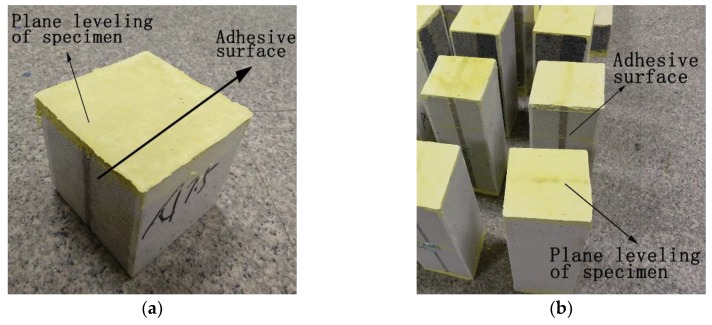
Specimen preparation for the compression test. (**a**) Cubic specimen; (**b**) Prism specimens.

There has been little research on the macroscale mechanical properties of TMFG, so a reasonable experimental design is very important. The adopted test methods were as for GB/T 5486-2008 standard [23] and concrete [24]. A compression section size of 100 mm × 100 mm was adopted in GB/T 5486-2008 standard [23]. The four-point flexural tests and compression tests were performed on specimens with six different batch compositions. The influences of the foaming agent SiC and the decoration surface on the properties of the materials were investigated. When group AD specimens (those with a decoration surface) were tested, we found that a bending occurred during the compression test due to the different strengths of the microcrystalline decoration surface and the microcrystalline foam glass parent material. Furthermore, to produce a decorative surface inside and outside of a wall in practical engineering, it is necessary to bond the material. Therefore, the cross section of two bonded 100 mm × 50 mm specimens was used in group AD. The thickness of the adhesive surface was 1–2 mm. The total size of the cross section of group AD was the same as that of group A in the same type of test. This section, shown in Figure 3, could ensure that no bending would occur during the compression test. The influence of the decorative surface was not considered in the shear and splitting tensile tests; therefore, only group A specimens were subjected to these tests. In each of the different tests, three specimens in each group were tested, and the results were averaged.

### 3.1. Four-Point Flexural Tests

**Figure 4:**
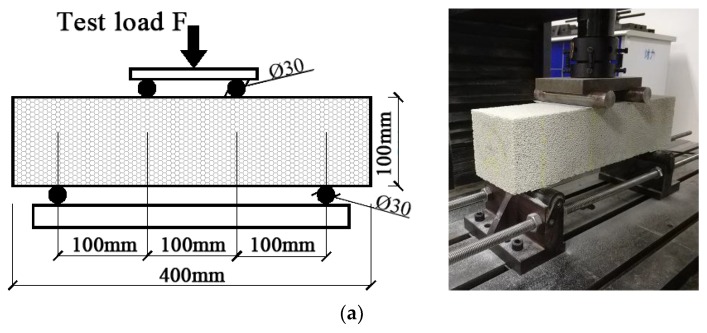

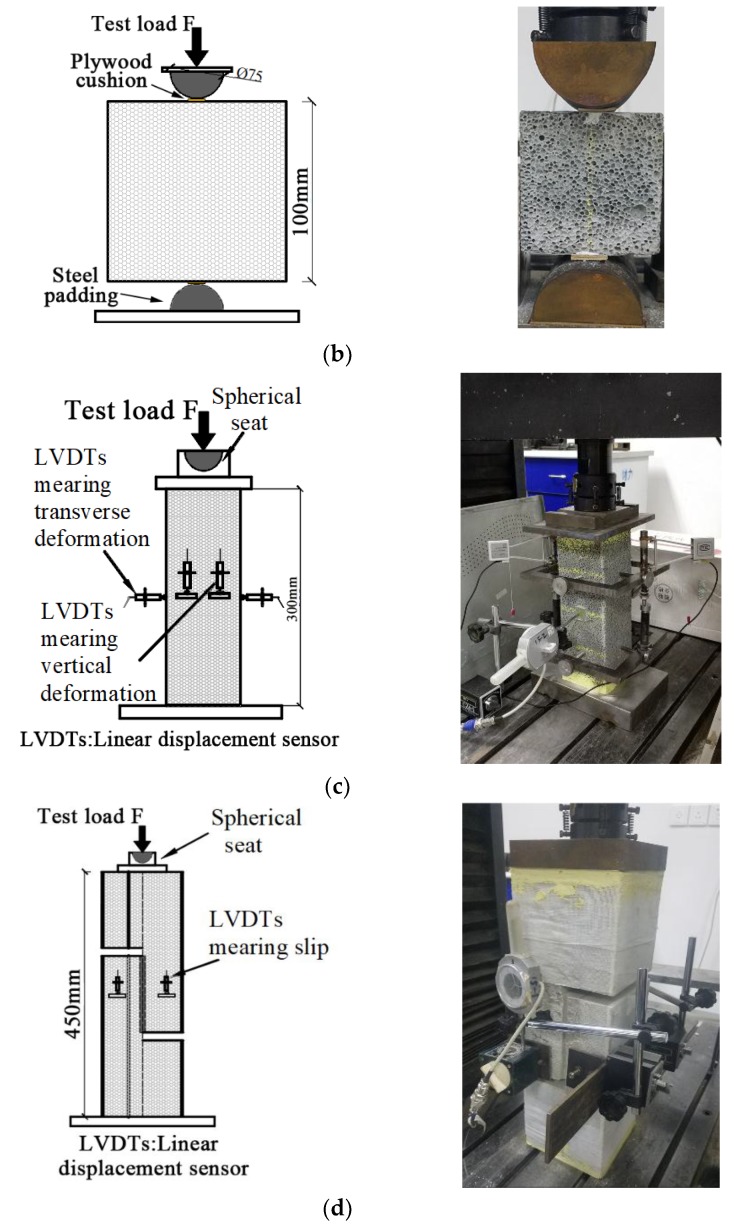
Test setups. (**a**) Four-point flexural tests; (**b**) Splitting tensile strength test; (**c**) Uniaxial compression test; (**d**) Shear Test.

**Figure 5:**
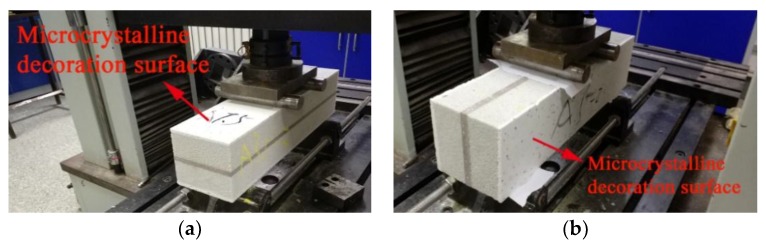
Two ways of placing the group AD specimens. (**a**) Decoration surface on the top (ADt); (**b**) Decoration surface on the side (ADs).

The size of the specimens for the four-point flexural tests was 100 mm × 100 mm × 400 mm (width × height × length). A total of 36 specimens was tested. The loading device is shown in Figure 4. We measured the influence of the microcrystalline decoration surface. Two different conditions were considered, i.e., the decoration surface was placed either on the top (ADt) or to the side (ADs), as shown in Figure 5. 

### 3.2. Splitting Tensile Strength Test

The size of the specimens for the splitting tensile strength test was 100 mm × 100 mm × 100 mm. The specimens were TMFG without the microcrystalline decoration surface. Nine specimens were tested, and the loading device is shown in Figure 4. The diameter of the steel padding was 75 mm. A plywood cushion was placed between the steel padding and the specimen. The width of the cushion should be 15–20 mm, the thickness should be 3–4 mm, the length should not be shorter than the side length of the specimen, and the plywood cushion should not be reused.

The splitting tensile strength can be calculated as follows:

(4) $$f_{\mathrm{st}}=\frac{2F_{\max}}{\pi A}$$

where $f_{\mathrm{st}}$ denotes the splitting tensile strength (MPa), $F_{\max}$ denotes the maximum test load (N), and A denotes the splitting area (mm^2^).

### 3.3. Compression Test

The size of the specimens for the cubic compression test was designed to be 100 mm × 100 mm × 100 mm, while that for the prism compression test was 100 mm × 100 mm × 300 mm (width × height × length). A total of 36 specimens were tested. The surface of TMFG has many pores, therefore, plaster was used to level the upper and lower surfaces of the specimens, as shown in Figure 3. The test device is shown in Figure 4.

### 3.4. Shear Test

**Figure 6:**
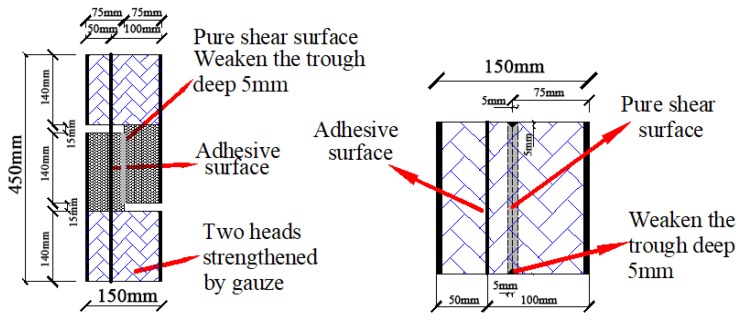
Cross section size of shear test specimen.

Considering the fragility of TMFG, the Z-shaped specimen method [24] was adopted for the shear test, with some improvements. Using gauze and building reinforcement resin, the non-shear parts were strengthened, and a shear-vulnerable surface was set up in the shear part to ensure pure shear failure. To ensure no bending would occur during the test, the specimen was made from TMFG with a microcrystal decorative surface, and the cross section was formed by bonding two blocks sized 50 mm × 150 mm × 450 mm and 100 mm × 150 mm × 450 mm. The test device is shown in Figure 4, and the section size of the specimen is shown in Figure 6. 

The shear strength can be calculated as follows:

(5) $$f_{s}=\frac{F_{\max}}{bh}$$

where $f_{s}$ denotes the shear strength (MPa), $F_{\max}$ denotes the maximum test load (N), b denotes the width of the pure shear surface (mm), and h denotes the height of the pure shear surface (mm).

## 4. Test Results

The result of the four-point flexural strength, compressive strength, splitting tensile strength, and shear strength tests were analyzed, and constitutive equations of TMFG are proposed in this paper.

### 4.1. Four-Points Flexural Test

**Figure 7:**
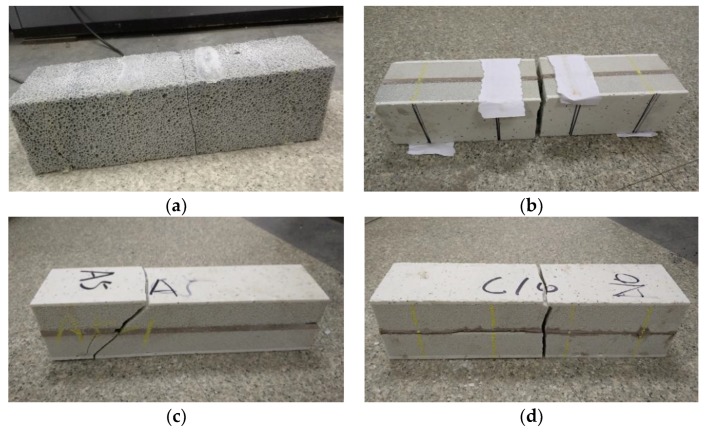
Destruction types in the specimen during the flexural test. (**a**) Group A; (**b**) Group Ads; (**c**) Group ADt1 and ADt2; (**d**) Group ADt3.

The flexure specimens from group A and group ADs produced sounds, indicating broken internal pores during the loading process, and vertical penetrating cracks in the middle part of the specimens were found. The destruction process without obvious signs was rapid, resulting in brittle failure. There were two forms of damage to the specimens in the group ADt. One was the shear slanting cracks that appeared in the largest shearing area. First, the microcrystalline foam glass parent metal was damaged, and, then, the microcrystalline decoration surface was destroyed in the largest shearing area. The damage process was rapid. The specimens ADt1 and ADt2 mainly exhibited this failure mode. The other form of destruction consisted of vertical cracks appearing in the largest moment area in the middle part of the specimen. The parent material was damaged first, and, then, the microcrystalline decoration surface was destroyed. This type of damage occurred in ADt3, as shown in Figure 7. The flexural strengths were calculated with Formula (6) and are compared in Table 4.

(6) $$f_{p}=\frac{F_{\max}L}{bh^{2}}$$

where $f_{p}$ denotes the flexural strength of the specimens (MPa), $F_{\max}$ denotes the maximum test load (N), L denotes the distances between the supports (mm), b denotes the specimen width (mm),and h denotes the specimen height (mm).

As shown in Table 4, the flexural strength increased with the decrease of the amount of SiC. The microcrystalline decoration surface also improved the flexural strength of TMFG; the flexural strengths of group ADt and ADs specimens were, respectively, 2.31 and 1.4 times greater than that of group A specimens. The orientation of the microcrystalline decoration surface greatly influenced the flexural strength; the flexural strength of group ADt specimens was approximately 1.64 times that of ADs specimens.

### 4.2. Compressive, Shear and Splitting Tensile Strength

**Figure 8:**
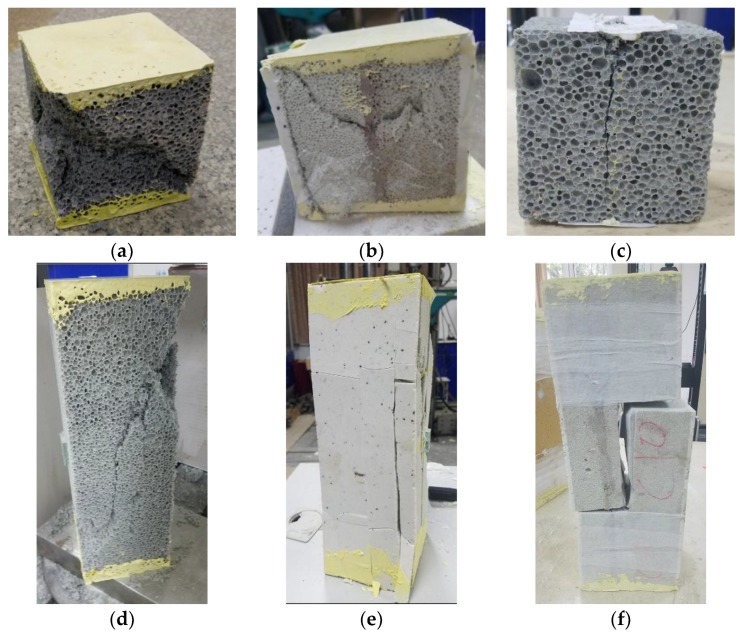
Failure types of the specimens. (**a**) Specimen of group A under cubic strength; (**b**) Specimen of group AD under cubic strength; (**c**) Splitting tensile failure for the group A specimen; (**d**) specimen of group A under prism strength; (**e**) Specimen of group AD under prism strength; (**f**) Specimen with shear failure.

**Figure 9:**
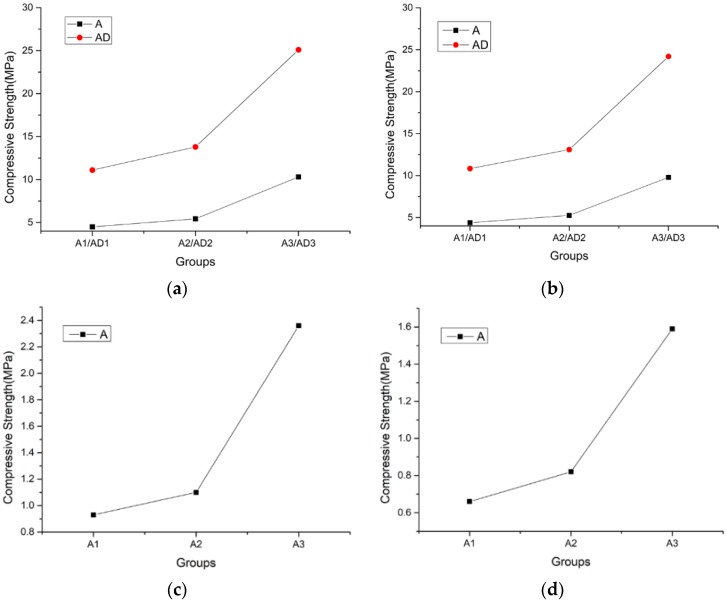
Variation in the strengths in groups A and AD. (**a**) Variation in cubic compressive strengths; (**b**) Variation in prism compressive strengths; (**c**) Variation in shear strengths; (**d**) Variation in splitting tensile strengths.

Figure 8 shows the specimens’ failure modes. The results of the compressive strength, shear strength, splitting tensile strength, and flexural strength tests, compared with those for other mechanical properties, are presented in Table 5; the unit of measure for all parameters was MPa. The strength changes for the different groups are shown in Figure 9.

**Figure 10:**
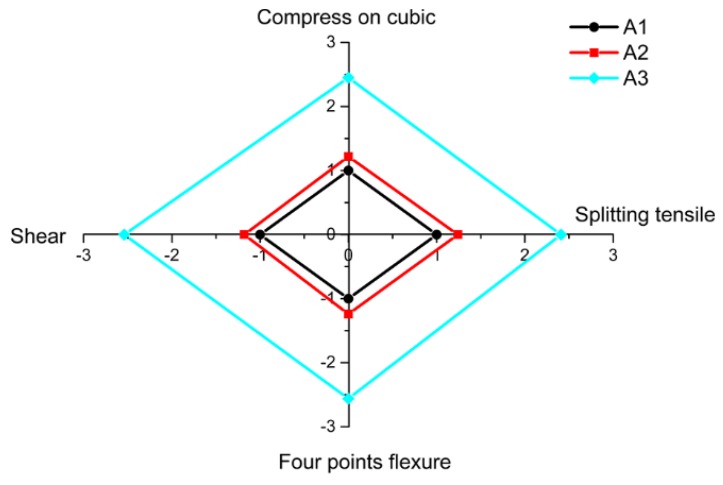
Strength of group A compared to that of A1.

As shown in Figure 9 and Table 5, the compressive strength, splitting tensile strength, shear strength, and flexural strength all increased with the decrease of the amount of SiC, due to a reduction of the foaming rate. The comparison of the cubic compressive strength, splitting tensile strength, shear strength, and flexural strength of the specimens in group A is shown in Figure 10. With the reduction in the amount of SiC, the improvement of the various mechanical properties was similar.

The various mechanical properties of A2 were about 1.22 times greater than those of A1, such as the compressive strength of A2 was 1.21 times that of A1, the splitting tensile strength of A2 was 1.24 times that of A1, the shear strength of A2 was 1.18 times that of A1, and the flexural strength of A2 was 1.24 times that of A1.

The various mechanical properties of A3 were about 2.45 times greater than those of A1, such as the compressive strength of A3 was 2.29 times that of A1, the splitting tensile strength of A3 was 2.41 times that of A1, the shear strength of A3 was 2.54 times that of A1, and the flexural strength of A3 was 2.56 times that of A1.

The cubic compressive strength of TMFG without the microcrystalline decoration surface was close to the prism compressive strength: the cubic compressive strength was about 1.04 times greater than the prism compressive strength. Further, the cubic compressive strength was about 4.7 times greater than the shear strength, about 6.64 times greater than the splitting tensile strength, and 2.92 times greater than the flexural strength.

The conversion relations are as follows:

(7) $$f_{\mathrm{cp}}=0.97f_{\mathrm{cc}}$$

(8) $$f_{s}=0.21f_{\mathrm{cc}}$$

(9) $$f_{\mathrm{st}}=0.15f_{\mathrm{cc}}$$

(10) $$f_{p}=0.34f_{\mathrm{cc}}$$

The compression strength of the specimens with the microcrystalline decoration surface was significantly higher than that of the specimens without it. The cubic strength of group AD was 2.48 times that of group A, and the prism strength of group AD was 2.47 times that of group A. Therefore, the microcrystalline decorative surface improved the flexural and compressive strength of TMFG.

### 4.3. Stress–Strain Constitutive Relations

**Figure 11:**
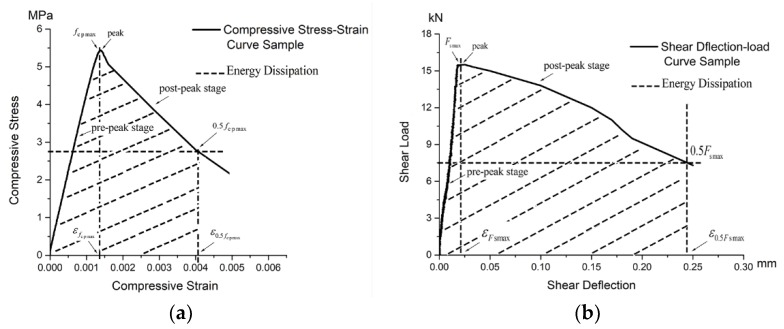
Representative curves for compressive stress and shear. (**a**) Representative curve for compressive stress; (**b**) Representative curve for shear load.

Figure 11 shows the representative compressive stress–strain and shear load–deflection curves. The loading process of TMFG was divided into two stages: a pre-peak stage and a post-peak stage (Figure 11). $f_{\mathrm{cpmax}}$, $0.5f_{\mathrm{cp}\max}$, $\varepsilon_{f\mathrm{cpmax}}$, and $\varepsilon_{0.5f\mathrm{cp}\max}$ in compression and $F_{s\max}$, $0.5F_{s\max}$, $\varepsilon_{F}{}_{\mathrm{smax}}$, $\varepsilon_{0.5F}{}_{s\max}$ in the shear load application are key parameters in the loading process. TMFG is brittle, and the later stage of the loading process resulted in abrupt failure; therefore, the curve in the post-peak stage was shallower.

**Figure 12:**
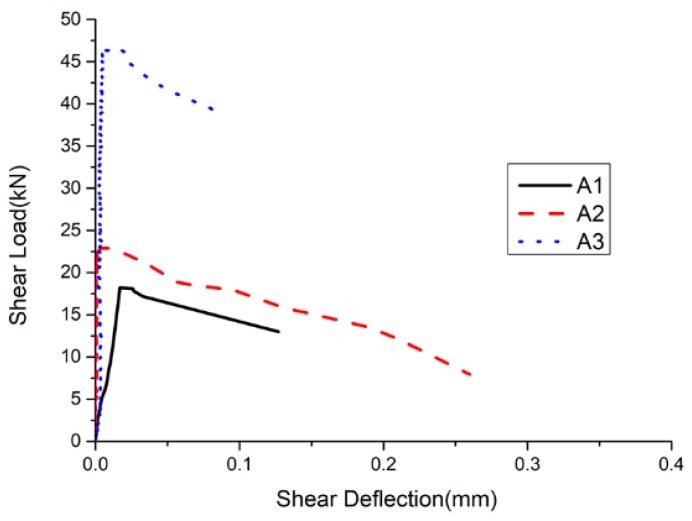
Shear load–deflection curves.

Figure 12 shows the shear load–deflection curve for three different batch compositions of group A. It can be seen that the reduction in the amount of SiC increased the shear strength of the specimens. However, the slope in the post-peak stage decreased, and the brittleness became more obvious.

**Figure 13:**
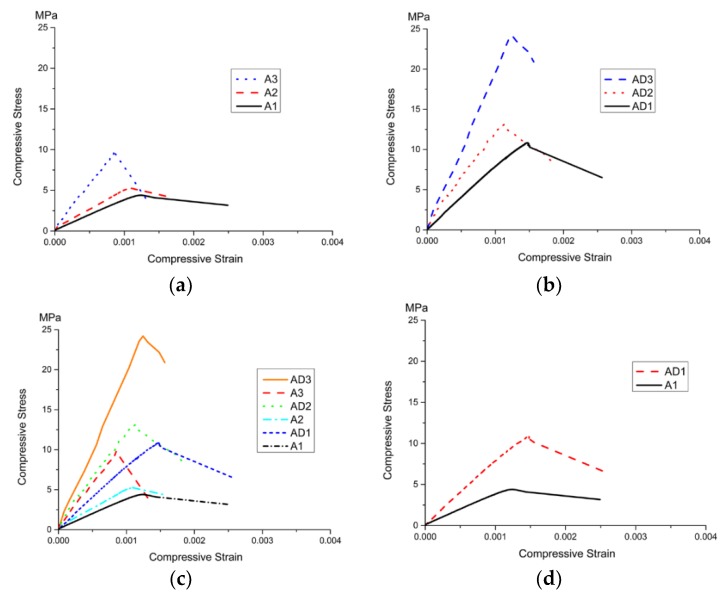

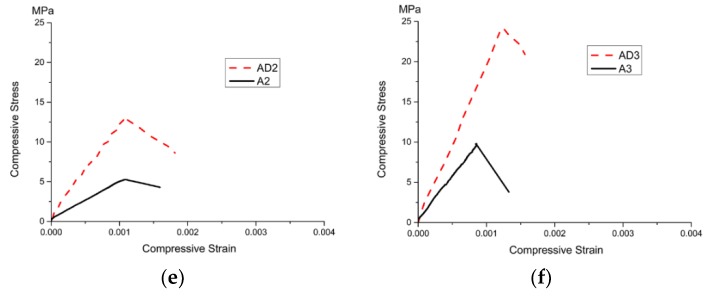
Compressive stress–strain curves. (**a**) Compressive stress–strain curves of Group A; (**b**) Compressive stress–strain curves of Group AD; (**c**) Compressive stress–strain curves of all specimens; (**d**) Comparison of stress–strain curves between A1 and AD1; (**e**)Comparison of stress–strain curves between A2 and AD2; (**f**) Comparison of stress–strain curves between A3 and AD3.

The compressive stress–strain curves of each group are shown in Figure 13. The compressive strength of the specimen increased with the reduction of the amount of SiC, but the slope of the curves in the post-peak stage decreased, and the brittleness became more obvious (Figure 13a–b). The microcrystalline decoration surface improved the compressive strength of the specimens, but the damage was more abrupt (Figure 13c–f).

**Figure 14:**
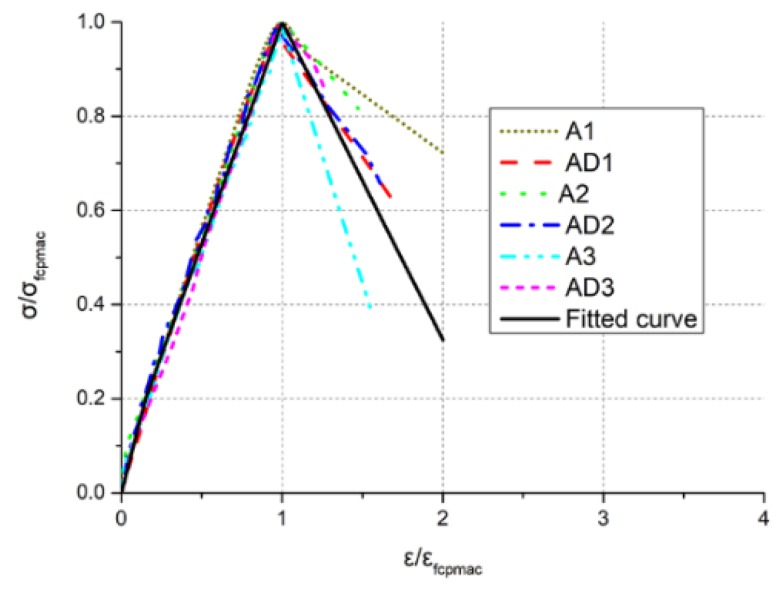
The dimensionless stress–strain curve and fitted curves.

In the dimensionless analysis of the compressive stress–strain curves, the ordinate is represented by $\sigma /\sigma_{c}$ (where the peak stress is $\sigma_{c}$), and the abscissa is represented by $\varepsilon /\varepsilon_{c}$ (where the peak strain is $\varepsilon_{c}$). The data were processed as shown in Figure 14.

There were initial microcracks inside the TMFG specimens. Because of the development of microcracks after stress application, the pre-peak stage curve would indicate stiffness changes in the initial stage; therefore, the 2-fold line formula was used to fit the pre-peak stage curve. The curve in the post-peak stage refers to the Hognestad concrete constitutive curve [25], and the residual stress was taken as $0.1f_{\mathrm{cpmax}}$. The 3-fold line Equation (11) was used to fit the loading process data for TMFG, and the fitting results are indicated by the solid line in Figure 14.

(11) $$y=\{\begin{array}{cc} \;1.33x & 0\leq x<0.15\\ 0.2+0.94(x-0.15) & 0.15\leq x<1\\ 1-0.9a(\frac{x-1}{x_{u}-1}) & x\geq 1 \end{array}$$

The fitting curve was in good agreement with the stress–strain curve, following the application of the segment adjustment parameter a=0.75 in Figure 14.

### 4.4. Modulus of Elasticity and Poisson’s Ratio

The modulus of elasticity and Poisson’s ratio are important indexes of a material. The slope of the pre-peak stage curve between $0.3f_{c}$ and $0.85f_{c}$ on the stress–strain curve was taken as the modulus of elasticity of the specimens, and shown in Table 6:

Table 6 shows that the modulus of elasticity increased with the reduction in the amount of SiC, and that the modulus of elasticity of the specimens with the microcrystalline decoration surface was higher than that of the specimen without the microcrystalline decoration surface. The modulus of elasticity of group AD was 2.71 times higher than that of group A.

Poisson’s ratio is an important index of a material’s lateral deformation. TMFG without the microcrystalline decoration surface is an isotropic material; therefore, the displacement meter was arranged on one of the symmetrical surfaces, and the transverse deformation of the specimen was recorded. For the specimens with the microcrystalline decoration surface, there was a small deformation in the direction of the decorative surface compared to the nondecorative surface; therefore, the displacement meter was arranged in the direction of the nondecorative surface. The data were processed using Formula (12) to obtain the Poisson’s ratio for each specimen, as shown in Table 7.

(12) $$v=\varepsilon^{\prime }/\varepsilon$$

where v denotes the Poisson’s ratio of the specimen, $\varepsilon^{\prime }$ denotes the transverse strain of the specimen, and ε denotes the longitudinal strain of the specimen.

The Poisson’s ratio tended to decrease with the increase in the compressive strength of TMFG, as shown in Table 7. Under the same conditions, because of the restraining effect of the decorative surface, the Poisson’s ratio of the specimens with the microcrystalline decoration surface was slightly smaller than that of the specimens without the microcrystalline decoration surface.

## 5. Discussion

### 5.1. Influence of the Physical Properties on the Mechanical Properties

**Figure 15:**
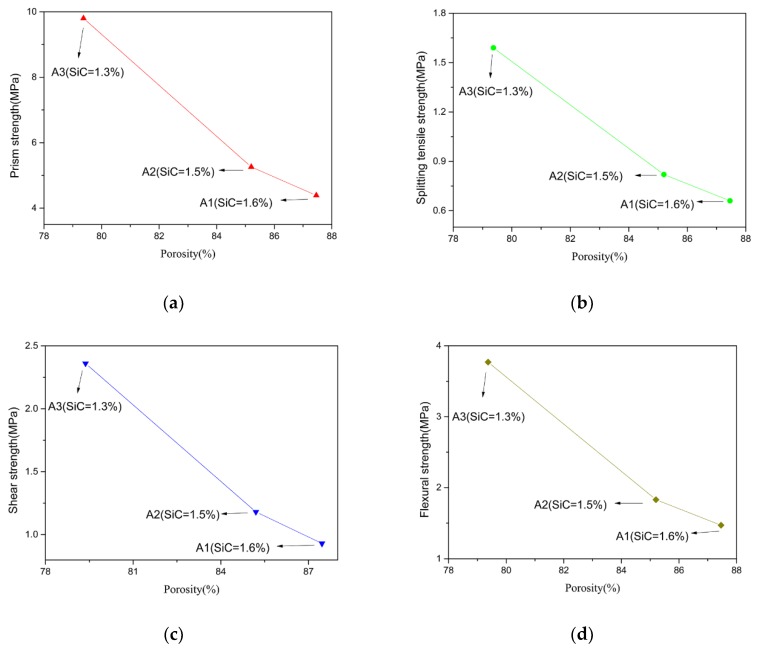
Comparison of the porosity and mechanical properties of group A1, A2, and A3 specimens. (**a**) Comparison of porosity and prism strength; (**b**) Comparison of porosity and splitting tensile strength; (**c**) Comparison of porosity and shear strength; (**d**) Comparison of porosity and flexural strength.

On the basis of the data of the physical properties in Section 2 and the results of the mechanical test in Section 4, the influence of the physical properties on the mechanical properties are discussed. The porosity and prism strength, splitting tensile strength, shear strength, and flexural strength of group A1, A2, and A3 were compared, as shown in Figure 15.

With the increasing of the amount of the foaming agent (SiC), the porosity increased. As the porosity was reduced, the prism compressive strength, splitting tensile strength, shear strength, and flexural strength increased, so that TMFG acquired better mechanical properties. Similar results were also found by F. Mear [2].

### 5.2. Evaluation of the Elastic Modulus

**Figure 16:**
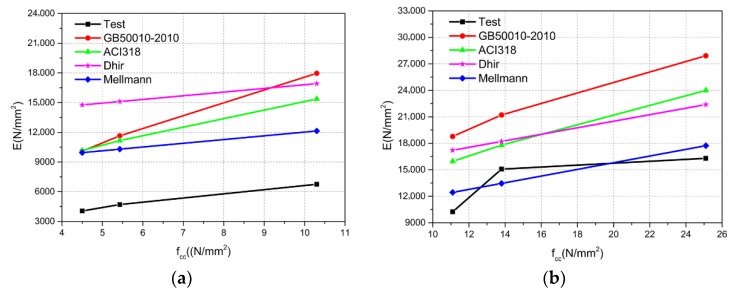
Modulus of elasticity from the test and results from each formula. (**a**) Group A; (**b**) Group AD.

Because of the scarcity of research on the macroscopic mechanical properties of microcrystalline foamed glass, relevant studies on the modulus of elasticity of concrete are here considered. Researchers have carried out many experimental studies on the modulus of elasticity of concrete, and their results have also been adopted to formulate various standards, such as the Chinese standard (GB50010-2010) [26] and the American standard (ACI318-08) [27]. Some researchers have derived relatively simple calculation formulas, such as those by Dhir [28] and Mellmann [29]. The modulus of elasticity was calculated by using these Formulas (13)–(16) and compared with the experimental data, as shown in Figure 16.

Code for the design of concrete structures (GB50010-2010)

(13) $$E_{C}=10^{5}/(2.2+34.7/f_{\mathrm{cc}})$$

Building code requirements for structural concrete (ACI318M-05)

(14) $$E_{C}=4789{\left(f_{\mathrm{cc}}\right)}^{1/2}$$

Calculation formula of Dhir

(15) $$E_{C}=13,100+370f_{\mathrm{cc}}$$

Calculation formula of Dhir Mellmann

(16) $$E_{C}=8242+378f_{\mathrm{cc}}$$

Figure 16 shows that most of the results from the theoretical formulas are different from and larger than the experimental results. As for the TMFG without the microcrystalline decoration surface, the trend of the curve is regular. The results of Group A and the calculation results were compared as shown in Table 8.

**Figure 17:**
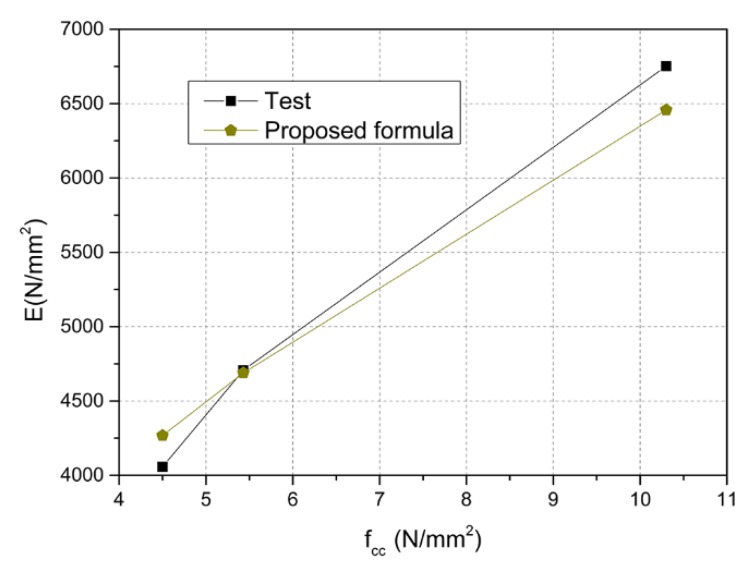
The elastic modulus calculated by the test and the calculation results obtained with the proposed formula.

As can be seen from Table 8, most calculation results are relatively large, and the maximum ratio between the experimental and the calculated results reached 3.12. Therefore, the existing design formula was not suitable for evaluating the elastic modulus of the TMFG. Based on the test results, Formula (17) was proposed to estimate the modulus of elasticity of Group A. The calculation results of Formula (17) were compared with the experimental results, as shown in Figure 17. Figure 17 shows that the calculation results of the proposed formula are in good agreement with the elastic modulus determined by the test, so Formula (17) could be used to estimate the modulus of elasticity of TMFG.

(17) $$E_{C}=2012{\left(f_{\mathrm{cu}}\right)}^{1/2}$$

## 6. Conclusions

(1) With the reduction of the amount of SiC, the porosity decreased, so that the mechanical properties improved. The improvement in the various mechanical properties was similar. The cubic compressive strength of TMFG without microcrystalline decoration surfaces was close to the prism compressive strength. The cubic compressive strength was about 1.04 times the prism compressive strength. The cubic compressive strength was about 4.7 times the shear strength, about 6.64 times the splitting tensile strength, and 2.92 times the flexural strength.
(2) The microcrystalline decorative surface improved the compressive and flexural strengths of TMFG. The compression strength of group AD was about twice that of group A. The flexural strengths of groups ADt and ADs were, respectively, 2.31 and 1.4 times greater than those of group A, and the orientation of the specimens significantly influenced the flexural strength of the specimens with the microcrystalline decoration surface. The flexural strength of group ADt was approximately 1.64 times that of ADs. The stress–strain curve of TMFG could be expressed by the 3-fold line equation.
(3) With the reduction of the amount of SiC, the modulus of elasticity of TMFG increased. The modulus of elasticity of TMFG was lower than the calculated value obtained using the concrete-specific formulas, and the highest ratio between the experimental and the calculated results was 3.12 times. A formula is proposed to estimate the modulus of elasticity of TMFG.
(4) With the increase in the compressive strength of TMFG, the Poisson’s ratio decreased. The Poisson’s ratio of the specimens with the microcrystalline decoration surface was slightly smaller than that of the specimens without the microcrystalline decoration surface.

## Figures and Tables

**Table 1:** Flexural strength (MPa). FG: foam glass; MFG: microcrystalline foam glass; TMFG: Tailing microcrystalline foam glass.

Material	Bulk Density (kg/m^3^)	Water Absorption Rate (vol %)	Thermal Conductivity (W/(m·K))	Compression Strength (MPa)
FG	200–400	0.8–11.0	0.10–0.13	1.5–3.0
MFG	933–1070	5.0–11.0	0.27–0.30	5–11
TMFG	300–600	1.8–2.4	0.075–0.38	4.0–12.5

**Table 2:** Batch composition. Group A: Specimens without microcrystalline decoration surface; Group AD: Specimens with microcrystalline decoration surface; Numbers 1–3: Three different batch compositions of the main raw materials.

Group	Main Raw Materials (%)			Foaming Agent (%)	Material of Decorative Surfaces (%)	
	Quartz	Waste Stone Residue	Waste Glass	SiC	Natural Gravel (Fine)	Natural Gravel (Coarse)
A1	75	10	15	1.6		
A2	80	10	10	1.5		
A3	80	10	10	1.3		
AD1	75	10	15	1.6	8	2
AD2	80	10	10	1.5	8	2
AD3	80	10	10	1.3	8	2

**Table 3:** Bulk density and porosity of TMFG. Group A: Specimens without microcrystalline decoration surface; Numbers 1–3: Three different batch compositions of the main raw materials.

Group	Bulk Density (kg/m^3^)	Porosity (%)	Water Absorption (%)
A1	340	87.46	2.41
A2	400	85.20	1.93
A3	560	79.37	1.83

**Table 4:** Flexural strength. Group A: Specimens without microcrystalline decoration surface; Numbers 1–3: Three different batch compositions of the main raw materials; ADt: Decoration surface on the top; ADs: Decoration surface on the side.

Group	1	Ratio	2	Ratio	3	Ratio	Mean Ratio	Placing Way
A	1.47 MPa	1	1.83 MPa	1	3.77 MPa	1	1	Top
ADt	3.04 MPa	2.06	5.68 MPa	3.10	6.66 MPa	1.77	2.31	Top
ADs	1.80 MPa	1.22	3.32 MPa	1.81	4.37 MPa	1.16	1.40	Side

**Table 5:** Compressive, shear, and splitting tensile tests results.

Groups	Cubic Strength $f_{\mathrm{cc}}$	Prism Strength $f_{\mathrm{cp}}$	Splitting Tensile Strength $f_{\mathrm{st}}$	Shear Strength $f_{s}$	Flexural Strength $f_{p}$
A1	4.50	4.39	0.66	0.93	1.47
A2	5.43	5.26	0.82	1.18	1.83
A3	10.30	9.80	1.59	2.36	3.77
AD1	11.1	10.74	-	-	-
AD2	13.8	13.1	-	-	-
AD3	25.1	24.2	-	-	-

**Table 6:** Modulus of elasticity of TMFG.

Groups	1	Ratio	2	Ratio	3	Ratio
A	4056.83	1.00	4707.10	1.00	6752.50	1.00
AD	10,244.00	2.53	15,071.50	3.20	16,291.67	2.40

**Table 7:** Poisson’s ratio of TMFG.

Group	1	2	3
A	1.41	0.88	0.44
AD	1.00	0.77	0.40

**Table 8:** Comparison of the test results and the results calculated from each formula.

Group	Test	GB50010	ACI318	Dhir	Mellmann
A1	4056.83	10,089.69	10,159.00	14,765.00	9943.00
A2	4707.10	11,640.87	11,159.50	15,109.10	10,294.54
A3	6752.50	17,956.76	15,369.63	16,911.00	12,135.40
Ratio	1.00	2.54	2.38	3.12	2.15
